# The CD4+ T Cell Response to Human Cytomegalovirus in Healthy and Immunocompromised People

**DOI:** 10.3389/fcimb.2020.00202

**Published:** 2020-05-19

**Authors:** Eleanor Y. Lim, Sarah E. Jackson, Mark R. Wills

**Affiliations:** Division of Infectious Diseases, Department of Medicine, Addenbrookes Hospital, University of Cambridge, Cambridge, United Kingdom

**Keywords:** human cytomegalovirus (HCMV), CD4+ T cell, solid organ transplant (SOT), hematopoietic stem cell transplant (HSCT), congenital CMV (cCMV)

## Abstract

While CD8+ T cells specific for human cytomegalovirus (HCMV) have been extensively studied in both healthy HCMV seropositive carriers and patients undergoing immunosuppression, studies on the CD4+ T cell response to HCMV had lagged behind. However, over the last few years there has been a significant advance in our understanding of the importance and contribution that CMV-specific CD4+ T cells make, not only to anti-viral immunity but also in the potential maintenance of latently infected cells. During primary infection with HCMV in adults, CD4+ T cells are important for the resolution of symptomatic disease, while persistent shedding of HCMV into urine and saliva is associated with a lack of HCMV specific CD4+ T cell response in young children. In immunosuppressed solid organ transplant recipients, a delayed appearance of HCMV-specific CD4+ T cells is associated with prolonged viremia and more severe clinical disease, while in haematopoietic stem cell transplant recipients, it has been suggested that HCMV-specific CD4+ T cells are required for HCMV-specific CD8+ T cells to exert their anti-viral effects. In addition, adoptive T-cell immunotherapy in transplant patients has shown that the presence of HCMV-specific CD4+ T cells is required for the maintenance of HCMV-specific CD8+ T cells. HCMV is a paradigm for immune evasion. The presence of viral genes that down-regulate MHC class II molecules and the expression of viral IL-10 both limit antigen presentation to CD4+ T cells, underlining the important role that this T cell subset has in antiviral immunity. This review will discuss the antigen specificity, effector function, phenotype and direct anti-viral properties of HCMV specific CD4+ T cells, as well as reviewing our understanding of the importance of this T cell subset in primary infection and long-term carriage in healthy individuals. In addition, their role and importance in congenital HCMV infection and during immunosuppression in both solid organ and haemopoietic stem cell transplantation is considered.

## Introduction

Over the last few decades research in both humans and murine models has clearly demonstrated that both the innate and adaptive branches of the immune response play a role in resolving both primary, reactivating and super-infections with cytomegalovirus (CMV). In particular, studies in transplantation patients (Sester et al., 2001; Einsele et al., 2002; Peggs et al., 2003; Gratama et al., 2008) and in adults with primary human CMV (HCMV) infections (Rentenaar et al., 2000; Gamadia et al., 2003; Lilleri et al., 2008b) have confirmed the vital role that HCMV specific CD4+ T cells play in controlling symptomatic disease. Here, we present a detailed overview of the evidence from many studies of the specific and direct anti-viral role that CD4+ T cells play in HCMV infections in the healthy and immunocompromised patients. In particular, we focus on the importance of both understanding and assessing the full functionality of CMV specific CD4+ T cells responses in patients to minimize the burden of CMV infection in transplantation and congenital infections.

### CD4+ T Cells, Their Activation and Role in Adaptive Immunity

CD4+ T cells are an important and multifaceted component of the adaptive immune response to viruses and other pathogens. In healthy adults, CD4+ T cells typically comprise the majority of T cells present. However, cytomegalovirus infection can lead to perturbation of the composition of circulating T cell populations (Chidrawar et al., 2009; Wertheimer et al., 2014). CD4 is a co-receptor that binds the Major Histocompatibility Complex (MHC) Class II molecules on antigen presenting cells (APC) that present peptides to the TCR present on the T cell (Glatzova and Cebecauer, 2019). MHC class II molecules comprise an α and β chain heterodimer which, when assembled, provide a peptide-binding cleft in which antigen is presented. In humans three gene loci encode the MHC class II molecules—HLA-DR, -DQ, and DP (Blum et al., 2013)—allowing for a wide diversity of peptides to be presented. MHC class II molecules are synthesized in the endoplasmic reticulum (ER) and transported to endosomes with an invariant chain present to stabilize the structure via the Golgi apparatus. Peptides generated by proteolysis of endocytosed proteins are exchanged for the fragment, Class II–associated Invariant Peptide (CLIP), which remains in the peptide binding cleft of the assembled MHC class II molecule within late endosomes. The loaded complex is then transported to the cell surface (Blum et al., 2013), hence allowing CD4+ T cells to recognize exogenously-derived proteins.

**Figure 1:**
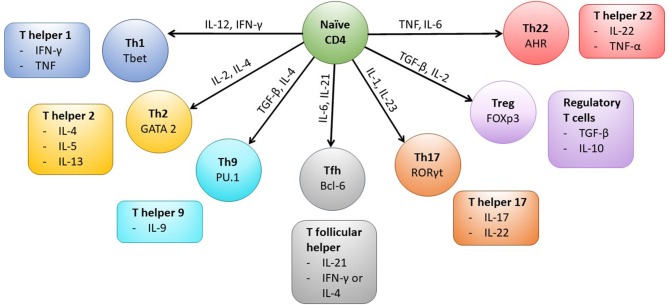
CD4+ T cell subsets and associated transcription factors and cytokines. Following activation of the CD4+ T cell cytokines present in the microenvironment (indicated on arrows) determine the type of effector cell that is induced by triggering expression of particular transcription factors (labeled in each cell subset). The typical cytokines secreted by each CD4+ T cell subset are also shown. Mature Th1 cells produce IFN-γ which can upregulate MHC Class I and II molecules on cells in the local microenvironment and the cells are anti-viral and protective against intracellular bacteria and fungi. Whereas, Th2 cells typically secrete IL-4, IL-5, and IL-13 and are active against extracellular parasites and implicated in allergy responses. Th9 cells are important in mediating anti-parasite immune responses, secreted IL-9 activates mast cells and increases basophil and eosinophil functions. T follicular helper cells (Tfh) are specialized to provide B cell help and assist in germinal center formation, mature Th17 cells aid in protection against extracellular bacteria and fungi. Treg cells are characterized by the expression of the transcription factor Foxp3 and help to control activation of the immune response, however Th22 cells have been shown to play a role in mediating immune responses in the skin.

Interaction of CD4+ T cells with MHC class II complexes on APCs results in formation of a complex known as an immunological synapse which precedes T cell activation. The formation of the synapse allows the clustering of various co-stimulatory molecules, including CD28 and CD40L, which are expressed by the T cell and are required for successful intracellular signaling and subsequent activation of the CD4+ T cell (Glatzova and Cebecauer, 2019). Following activation, the CD4+ T cell population expands and then typically contracts before establishing a memory population. The generation of long lived antigen specific memory CD4+ T cells involves the integration of multiple cellular and cytokine processes (Kara et al., 2014; Nguyen et al., 2019). CD4+ memory T cells can be subdivided into a number of different functional subsets (Figure 1), which includes T helper 1 (Th1) and T regulatory (T_reg_) cellular types. The generation of the different subsets is a result of the location of the CD4+ T cell, the local cytokine environment, and expression of cellular transcription factors. For example, differentiation into the Th1 subset, which is characterized by production of anti-viral cytokines such as IFN-γ, is as a result of exposure to IL-12, IFN-γ, and expression of the transcription factor T-bet (Zhu et al., 2010; Nguyen et al., 2019). CD4+ T cell memory and effector populations can also be defined according to their differentiation status, which is indicated by the expression or loss of expression of various cell surface markers. Common memory subsets include central memory (T_CM_), effector memory (T_EM_), CD45RA re-expressing effector memory cells (T_EMRA_) and Tissue resident memory (T_RM_) subsets (Nguyen et al., 2019). CMV-specific CD4+ T memory recall cell responses have typically been shown to be of a differentiated memory cell phenotype, where downregulation of co-stimulatory molecules CD27 and CD28 and expression of CD57 and re-expression of CD45RA are observed (van Leeuwen et al., 2004; Weekes et al., 2004; Fletcher et al., 2005; Casazza et al., 2006; Lilleri et al., 2008b; Libri et al., 2011; Dirks et al., 2013). Memory CD4+ T cell populations also express chemokine receptors and integrin-related proteins, which allow homing to specific tissue sites (Nguyen et al., 2019). For instance, CMV-specific memory CD4+ T cells have been shown to express CX3CR1, enabling homing of these cells to activated vascular endothelium (Pachnio et al., 2016).

### CD4+ T Cells and Anti-viral Immunity

The roles that CD4+ T cells fulfill in anti-viral immune responses can broadly be divided into 3 categories: recruitment of lymphoid cells to sites of infection, mediating expansion or function of other effector cells, or providing direct anti-viral effects through cytokine production or cell-mediated cytotoxicity. The classic view of CD4+ T cells is as a helper cell. In anti-viral responses they help recruit CD8+ T cells to sites of infection by promoting engagement of CD8+ T cells with dendritic cells via chemokines such as CCL3 and CCL4. They can also facilitate entry of naïve CD8+ T and B cells to draining lymph nodes and recruit innate or antigen-specific effectors to sites of viral replication via production of IFN-γ and local chemokine secretion. CD4+ T cells can also mediate expansion and function of both B cells and CD8+ T cells. Binding of antigen on CD4+ T cells initiates expression of CD40L, which engages CD40 on B cells and induces proliferation and differentiation of B cells, initially in extra-follicular foci and then in germinal centers of lymph nodes, resulting in production of antibody-producing plasma cells and memory B cells. With CD8+ T cells, CD4+ T cells have been shown to facilitate development of memory CD8+ T cells via various mechanisms, such as through downregulation of TNF-related apoptosis-inducing ligand (TRAIL) expression, generation of cytokines such as IL-2, or direct ligation of CD40 on naïve CD8+ T cells by CD40L on CD4+ T cells (Sant and McMichael, 2012; Swain et al., 2012).

Finally, there has been increasing evidence of a role of CD4+ T cells in antiviral immunity that is independent of their helper function through two distinct mechanisms: production of cytokines IFN-γ and TNF, and through direct cytolytic actions via perforin- and Fas-dependent killing (Juno et al., 2017). In particular, these cytotoxic T cells have been described to emerge after CMV infection (van Leeuwen et al., 2004) and have demonstrated a capability to lyse CMV antigen-expressing target cells *in vitro* (van Leeuwen et al., 2006). The majority of CD4+ T cells produced in response to viral infection are of the T-helper 1 subtype, producing IFN-γ and expressing the transcription factor T-bet (Caza and Landas, 2015). This has also been observed following primary CMV infection (Rentenaar et al., 2000). However, other functional subsets are also involved in anti-viral immunity. T follicular helper cells, characterized by their expression of the chemokine receptor CXCR5 and transcriptional repressor Bcl6, produce IL-21 which facilitates germinal center B cell selection and differentiation of activated B cells that provide long-term antibody-mediated protection against viral pathogens (Hale et al., 2013; Hale and Ahmed, 2015). Regulatory T cells (Tregs), identified by expression of Foxp3 and CD25 on their cell surface, limit immunopathology in chronic viral infections (Karkhah et al., 2018). Tregs that develop in the thymus are termed natural Tregs, while those that develop in peripheral lymphoid organs are termed inducible Tregs (iTregs). In the context of anti-viral responses to CMV, CMV-specific iTregs were found to be increased in older women and may attenuate the chronic vascular injury caused by CMV (Terrazzini et al., 2014).

### The Role of CD4+ T Cells Against HCMV Infection in the Healthy

Primary HCMV infection in the immunocompetent host is usually asymptomatic and may manifest as a viral syndrome, occasionally accompanied by end-organ involvement—commonly hepatomegaly, splenomegaly and lymphadenopathy. In immunocompetent individuals, the innate and adaptive arms of the immune system are capable of limiting lytic viral replication and preventing end-organ disease (Crough and Khanna, 2009) resulting in a largely self-resolving mononucleosis-like illness, although the virus then establishes a lifelong persistent infection through latency with periods of reactivation, during which productive lytic infection occurs (Sinclair and Poole, 2014). Rarely, HCMV infection in adults with effective immune responses does cause severe disease. The immune response in these individuals are typically characterized by large expansions of NK cell and T cell populations, particularly CMV-specific CD8+ T cells (Riou et al., 2017). CMV-specific CD8+ T cell populations have been studied extensively and are an essential component of effective immune control of CMV infection, as studies in transplant patients have clearly shown that recovery of the CMV specific CD8+ T cell response is crucial to successful protection against CMV disease (Tormo et al., 2010a,b, 2011). Indeed, the earliest studies investigating the effectiveness of adoptive T cell transfer therapy revealed that patients receiving *ex vivo* expanded CMV specific CD8+ T cells are protected from both primary and reactivating infection (Riddell et al., 1992; Walter et al., 1995; Einsele et al., 2002; Peggs et al., 2003). In healthy HCMV sero-positive adults there has been found to be a high frequency of CMV-specific memory T cell populations, with epitopes derived from pp65 and IE1 regularly reaching 5–10% of total CD8+ T cells in peripheral blood (Khan et al., 2002a,b; Sylwester et al., 2005). Another characteristic of the expanded CMV specific memory CD8+ T cell populations is their highly differentiated phenotype, including a large proportion of cytotoxic effector memory cells which have re-expressed CD45RA (Jackson et al., 2011, 2014).

Originally the role of CD4+ T cells in mounting anti-CMV responses was presumed to be a supportive one, enhancing CD8+ T cell responses to the virus (Tormo et al., 2011). However, multiple studies in transplant settings and infants infected with CMV show that poorer CD4+ T cell responses result in a prolonged course of viral shedding and more severe disease (Sester et al., 2001; Einsele et al., 2002; Peggs et al., 2003; Tu et al., 2004; Gratama et al., 2008). Studies of the role of CMV-specific CD4+ T cells during acute CMV infection in healthy adults have mainly been conducted in pregnant women cohorts, these have revealed that at early time points post infection responses to gB and pp65 CMV proteins are the dominant responses (Mele et al., 2017). However, the frequency of CMV specific CD4+ T cells to primary infection are lower compared to memory responses (Antoine et al., 2012; Fornara et al., 2016, 2017; Mele et al., 2017), responding CD4+ T cells have lower functional avidity (Antoine et al., 2012) and express higher levels of immune checkpoint proteins such as PD-1 compared to CMV specific CD4+ T cell memory responses (Antoine et al., 2012; Mele et al., 2017; Riou et al., 2017). Whereas, recall memory CD4+ T cell populations in CMV seropositive donors are characterized by expanded highly specific effector memory populations (Bitmansour et al., 2002) with multiple functions (Casazza et al., 2006). Although the frequency of CMV specific CD4+ T cell memory responses are expanded compared to those established at the time of infection, there is very little evidence of continual accumulation, so called “memory inflation,” of CMV specific CD4+ T cells over time in humans (reviewed in Jackson et al., 2019).

#### HCMV Antigen Specificity of CD4+ T Cells

Initially, studies to identify HCMV-specific CD4+ T cells used lysate derived from HCMV infected fibroblast cells to stimulate the antigen-specific response (Lindsley et al., 1986; Sester et al., 2002; Pourgheysari et al., 2007, 2009). Subsequently, studies of the CD8+ T cell repertoire identified multiple peptides that were most frequently recognized by HCMV-specific CD8+ T cells (Kern et al., 1999; Wills et al., 2002; Elkington et al., 2003; Gibson et al., 2004). Among the most commonly recognized were pp65 and IE-1, although some structural, early/late antigens, and HCMV-encoded immunomodulators were also identified (such as pp28, pp50, gH, gB, US2, US3, US6, and UL18) (Elkington et al., 2003). This was also used to guide studies identifying CD4+ T cells that were HCMV-specific (Weekes et al., 2004). An in depth study of T cell responses to 213 HCMV open reading frames (ORFs) found that CD4+ T cells recognize proteins from up to 125 different ORFs. In particular, CD4+ T cells recognized immediate-early (IE) gene products by 2.3-fold over their representation in the HCMV genome, and there was also preferential recognition of primary immune evasion proteins and viral tegument and glycoproteins (Sylwester et al., 2005). Recognition of HCMV glycoproteins by CD4+ T cells has also been reported in a number of other studies (Crompton et al., 2008; Pachnio et al., 2015, 2016).

Measurement of the functional capability of these cells has also evolved. Most studies have measured intracellular cytokine production, predominantly IFN-γ, to determine if the CD4+ T cells were specific for HCMV (reviewed in Jackson et al., 2011). More recently, work has demonstrated a functional capability of these cells *in vitro*, where autologous HCMV-specific CD4+ T cells (identified by upregulation of activation markers CD40L and 4-1BB above the background response) were shown to be able to restrict viral dissemination in monocyte-derived dendritic cells (Jackson et al., 2017). In addition, CD4+ T cells from a cohort of healthy seropositive donors were also found to recognize latency-associated viral genes UL138 and LUNA (latency-associated unidentified nuclear antigen), and the T-cell response to these antigens included secretion of cIL-10, an immunosuppressive cytokine that may function to suppress anti-viral immune responses (Mason et al., 2013). Suppressive CMV-specific CD4+ T cells that secrete IL-10 or have a phenotype of a regulatory cell (T_reg_) have been identified in other studies (Tovar-Salazar et al., 2010; Schwele et al., 2012; Terrazzini et al., 2014; Clement et al., 2016) and these cells likely play an important role in controlling the immune response to CMV in reactivating disease in particular. Follicular helper T cells (Tfh) that are CMV-specific for the glycoprotein pentameric complex (gH/gL/pUL128L) increase in numbers during the early phase of infection resulting in a rise in neutralizing antibodies, once the virus is cleared Tfh numbers decrease but glycoprotein specific Tfh CD4+ T cells are maintained over time (Bruno et al., 2016). CMV-specific CD4+ T cells, identified either by upregulation of activation markers or using MHC class II tetramers, have also been shown to have cytotoxic capacity, measured via surrogate markers such as expression of CD107a (a marker of degranulation), detection of intracellular perforin and granzyme molecules or via cytotoxicity assays including chromium release assays (Gamadia et al., 2004; van Leeuwen et al., 2004, 2006; Crompton et al., 2008; Mason et al., 2013; Pachnio et al., 2015, 2016; Jackson et al., 2017). This suggests that CMV-specific CD4+ T cells have the ability to kill CMV infected cells.

### HCMV Immune Evasion of CD4+ T Cell Responses

The HCMV genome encodes multiple evasion proteins during the course of infection that allows the virus to modulate intrinsic, innate and adaptive immune responses (Wills et al., 2015), the end result of this being the persistence of active primary infection viremia even in the immunocompetent host, which is accompanied by virus excretion for months (in adults) or even years (in children). Of particular relevance to this review of CD4+ T cell responses to HCMV is the observation that persistent shedding of virus into urine and saliva is associated with a lack of CD4+ T cell response in healthy children (Tu et al., 2004).

#### Evasion via Downregulation of MHC Class II Proteins by US2 and US3

**Figure 2:**
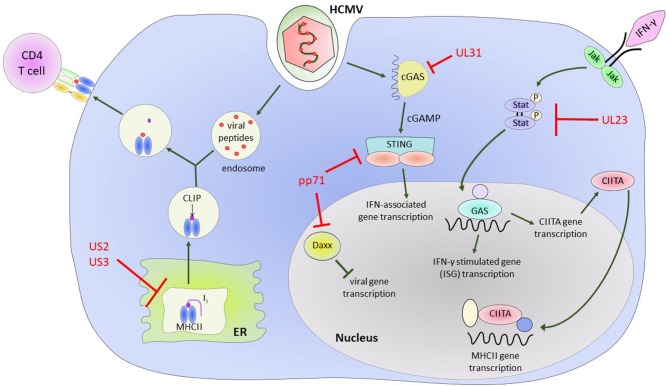
HCMV encoded proteins which help to evade CD4+ T cell mediated immune responses. Illustrating the impact of US2 and US3 on the MHC Class II protein presentation pathway and the effect of various HCMV encoded proteins on Class II Transcriptional activator (CIITA) and interferon gamma (IFN-γ) signaling pathways and IFN-γ stimulated gene (ISG) transcription.

Early work characterizing essential and non-essential genes of HCMV found that infection led to downregulation of MHC class I molecules on the surface of infected cells (Barnes and Grundy, 1992; del Val et al., 1992; Beersma et al., 1993; Gilbert et al., 1993; Yamashita et al., 1993). The US1-US11 region of the HCMV genome encodes at least 4 proteins, US2, US3, US6, and US11 that can independently interfere with the stability, assembly or export of MHC class I and II molecules (Johnson and Hill, 1998; Ploegh, 1998). US2 has been shown to affect the MHC class II processing pathway, specifically by binding to MHC class II-α chains and assembled MHC class II-α/β/Ii complexes, leading to their degradation (Tomazin et al., 1999). US3 alters assembly of MHC class II complexes by binding HLA-DR (but not HLA-DM) proteins before or during assembly of α/β complexes in the ER, preventing the binding of the invariant chain. This leads to mislocalization of these complexes to other post-Golgi compartments and results in the reduction of antigen presentation in US3-expressing cells (Hegde et al., 2002) (Figure 2).

#### Evasion via Downregulation of Class II Transcriptional Activator and Modulating the Effects of IFN-γ

Interferon-γ (IFN-γ) upregulates MHC class II molecules in cells constitutively expressing MHC class II, such as B cells, dendritic cells, and professional antigen presenting cells (APCs). However, it is also able to induce MHC class II expression in cells that do not constitutively express MHC class II, such as epithelial cells and fibroblasts, via the MHC class II transactivator gene (CIITA) (Steimle et al., 1994). The mechanism of how this occurs is not fully elucidated, however it involves regulation of a number of signaling pathways and transcription factors in a cell specific manner. Binding of IFN-γ to its cell-surface receptor activates the protein tyrosine kinases Jak1 and Jak2, and activation of these Jak kinases phosphorylates the tyrosines of the cytoplasmic transcription factor Stat1, and translocates it to the nucleus. Stat1 then binds directly to the IFN-γ-activation site (GAS) element of CIITA. The CIITA promoter region also includes an interferon regulatory factor (IRF)-1 binding site and binding of both these regions are essential for activation by IFN-γ (Muhlethaler-Mottet et al., 1998). Activation of CIITA leads to the assembly of a MHC class II enhanceosome, triggering a cascade of events that ends in autophosphorylation of CIITA and allows transcription of MHC class II genes to initiate (Devaiah and Singer, 2013). In macrophages the transcription factor NFAT5 is required for expression of the CIITA and MHC class II molecules, but this is not the case for dendritic cells and B cells (Buxade et al., 2018).

The HCMV genome encodes for a number of proteins that assist in modulation of the effects of IFN-γ (Goodwin et al., 2018) and directly modulate CIITA transcription. In Langerhans cells, a dendritic cell subset, HCMV infection results in a decrease in constitutive expression of CIITA (Lee et al., 2011). Further evidence in a transfected cell line model system showed that CMV downregulates MHC class II expression on the cell surface via regulation of CIITA and independently of known CMV Class II modulators US2 and US3 (Cebulla et al., 2002). Recently, it has also been shown in kasumi-3 cells, a myeloid lineage tumor cell line, that reduction in endogenous expression of MHC class II is as a result of decreased CIITA transcription (Sandhu and Buchkovich, 2020). UL23 binds to the Stat effector molecule N-myc, preventing proper activation and translocation of the Stat1 homodimers required for IFN-γ signaling (Feng et al., 2018), while UL31 preferentially binds the cytosolic DNA sensor cGAS in a manner that results in inhibition of interferon-associated gene transcription (Huang et al., 2018). The tegument protein pp71 binds Daxx, a Death-domain associated protein, and targets it for degradation, resulting in an inhibitory effect on induction of downstream antiviral genes (Cantrell and Bresnahan, 2006; Hwang and Kalejta, 2007; Lukashchuk et al., 2008). It has also been demonstrated that pp71 can negatively regulate the signaling role of STING (Stimulator of Interferon Genes) by inhibiting its translocation to the nucleus and preventing recruitment of accessory proteins to the complex (Fu et al., 2017) (illustrated in Figure 2). The end result of all these modulations is a decrease in transcription of downstream interferon-γ-associated genes, which, among other effects, results in decreased expression of MHC class II on the surface of infected cells and a decreased ability to present antigen via the MHC class II antigen presentation pathway.

## HCMV Specific CD4+ T Cells Responses in Immunocompromised and Immunonaïve Patients

As already discussed, studies in transplant patients have shown the essential role that the CMV-specific CD4+ T cell response plays in successful resolution of active CMV infection in this setting. Many of these studies were performed due to the significant morbidity and mortality caused by opportunistic CMV infection and reactivation in immunocompromised and immunonaïve patients. Therefore, as well as informing our understanding of how the immune response to CMV works, these studies have revealed much about the role of CD4+ T cell responses in specific transplantation and congenital environments and the possible manipulation of these responses to improve clinical outcomes.

### CD4+ T Cell Responses to HCMV in Solid Organ Transplant Recipients

Cytomegalovirus is the most common viral opportunistic infection in solid organ transplant (SOT) recipients, with the risk of infection or reactivation being stratified according to the CMV sero-status of the donor and recipient. An organ donation from a sero-positive donor to a sero-negative recipient (D+/R–) carries the highest risk, a sero-positive recipient (R+) is at intermediate risk, and D-/R- transplants are at lowest risk (Kowalsky et al., 2013). Other factors affecting risk of CMV reactivation or disease include the type of organ transplanted, with lung and small intestine transplant recipients having the highest risk, while liver and heart recipients are at an intermediate risk and kidney recipients are at the lowest risk of CMV infection (Humar et al., 2009). The reasons for this stratification are likely related to the amount of immunosuppression required, and the latent viral load present in these organs (Meesing and Razonable, 2018). In addition, use of antilymphocyte antibody induction agents also increase risk of reactivation (Preiksaitis et al., 2005).

In 2001, it was demonstrated that in the first months after kidney transplantation clinical symptoms of CMV disease were preceded by a decrease in CMV-specific CD4+ T cell frequencies (Sester et al., 2001). Subsequently it was found that significantly lower CD4+ T cell responses (measured by IFN-γ production) to pp65 were associated with concurrent CMV replication in sero-positive recipients, suggesting that CMV-pp65 CD4+ T cell responses above 0.03% in PBMCs of kidney transplant patients under stable immunosuppression were associated with lower risk of concurrent and future CMV replication for the following 8 weeks (Egli et al., 2008). In liver transplant recipients, the data is more heterogenous. A study of 17 high-risk liver transplant patients (D+/R–) found IFN-γ production by CD4+ T cells following CMV lysate stimulation in all patients, but no significant association between the presence of these CMV-specific IFN-γ-producing CD4+ T cells and development of CMV viremia in this cohort (La Rosa et al., 2007). However, another study involving 29 liver transplant patients found that CD4+ T cells producing IFN-γ, IL-2 or both cytokines in response to a peptide mix containing pp65, IE1, and CMV lysate occurred at a lower frequency in recipients who subsequently develop viremia (Nebbia et al., 2008).

In studies involving SOT where the recipient is CMV sero-positive the results are more consistent. A study involving 38 SOT CMV sero-positive recipients showed that patients with a higher number of HCMV-specific CD4+ T cells detected prior to transplantation were more likely to have earlier immune restoration and less likely to have HCMV infections requiring anti-viral treatment (Gerna et al., 2006). Subsequent studies involving larger cohorts of SOT recipients have corroborated these observations, showing that reconstitution of HCMV–specific CD4+ and CD8+ T cell responses were required to control infection, whereas patients who only regained CD8+ T cell responses were not (Gerna et al., 2011; Lilleri et al., 2018). Recently it has been shown that a reduction in the size of CMV specific CD4+ T cell responses measured using a diagnostic flow cytometry test is more predictive of CMV events occurring than the reduction in CD8+ T cells in transplantation patients (Rogers et al., 2020).

The protective nature of the CMV specific CD4+ T cell responses to multiple CMV proteins was tested by using CMV-infected autologous *in vitro* derived dendritic cells as stimulation. This was found to be more effective at predicting protection from disease than using only pp65 or IE proteins as stimulus in a small cohort of SOT patients (Lilleri et al., 2007b). This observation is supported by more recent work in a cohort of D+/R- liver transplant patients which compared the use of prophylactic vs. pre-emptive anti-viral therapy. Both patients groups had similar CD4+ T cell responses to CMV proteins pp65 and IE-1 despite 40% of the anti-viral prophylaxis group developing late stage CMV disease (Limaye et al., 2019).

In summary, the presence of CMV-specific CD4+ T cells is associated with lower risk of CMV disease. However, studies regarding the role of CD4+ T cells in SOT recipients have largely focused on using this as a predictive tool (San-Juan et al., 2015; Burton et al., 2018, 2019; Lilleri et al., 2018), and it is important to consider that these studies have relied on the production of IFN-γ by CD4+ T cells in response to peptide stimulation or virally infected dendritic cells as predicting the effectiveness of CMV specific CD4+ T cell responses. This disregards other potential anti-viral functions of the CD4+ T cells, such as cytotoxic capacity or other secreted anti-viral factors, as a predictive tool of the likelihood of CMV disease in solid organ transplantation.

### CD4+ T Cells and Haematopoietic Stem Cell Transplantation

In haematopoietic stem cell transplant (HSCT) recipients, the highest risk of CMV viremia and disease occurs in the reactivation of latent infection in R+ patients due to the ablation of their existing CMV specific T cell response. In particular, D-/R+ recipients are at a higher risk than D+/R+ patients, as reactivation of latent disease in the sero-positive recipient will appear as a primary CMV infection to the naïve lymphocytes transplanted from the sero-negative donor (Hebart and Einsele, 2004; van der Heiden et al., 2018b).

#### Use of CD4+ T Cell Response to Predict Risk of HCMV Viremia or Disease and the Relationship to End-Organ Disease

Early observational studies of haematopoietic stem cell transplant patients focused on examining the association of absolute CD4+ T cell recovery in these patients with the risk of development of HCMV viremia and end-organ disease, with the aim of predicting those at risk for CMV reactivation and disease. A study of allogeneic bone marrow transplant (BMTs) patients showed that a decrease in the lymphocyte count to <300 cells/μl occurred among patients who developed CMV disease, and that a decrease of CD4+ T cells numbers to <100/μl, 49 days following BMT was 100% predictive for the development of CMV disease in patients. In addition, persistent CD4 lymphopenia was only observed in patients who died of CMV disease (Einsele et al., 1993). A subsequent study of a cohort of 71 recipients of T-cell-depleted BMTs showed that life-threatening opportunistic infections occurred exclusively in patients whose CD4+ counts were <200 cells/μl and were fatal in all patients except those receiving donor leukocyte infusions (Small et al., 1999). These findings are also applicable for end-organ disease—a comparison of 2 BMT recipients who developed CMV retinitis with 14 patients who did not showed that the retinitis patients had significantly lower CD4+ T cell counts (Kuriyama et al., 2001).

Whilst the recovery of CD4+ T cell numbers post HSCT is an important measure in understanding the role they play in CMV disease in these patients, it is also important to track and measure the development of HCMV-specific CD4+ T cell responses and this association with the risk of HCMV reactivation and overt disease occurring. A study of 48 allogeneic HSCT recipients found that patients who had developed a CMV-specific CD4+ T cell response by 4 weeks post-transplant, measured by IFN-γ production following stimulation with CMV antigens, had lower peak CMV viral loads compared to patients with negative stimulation results (Avetisyan et al., 2006). In a pediatric allogeneic HSCT recipient cohort, the presence of 1 HCMV-specific CD4+ T-cell/μl of blood was protective against recurrent episodes of HCMV viremia (Lilleri et al., 2006). The same group also examined an adult allogeneic HSCT patient cohort, finding that the same cut-off level of 1 HCMV-specific CD4+ T-cell/μl of blood was able to identify patients who could spontaneously control HCMV infection in the absence of treatment (Lilleri et al., 2008a). Studies from other transplant centers have also seen similar results in minimum numbers of CMV-specific CD4+ T cells predictive of preventing CMV viremia or disease (Solano et al., 2008; Pourgheysari et al., 2009; Tormo et al., 2011).

Most studies examining the role of CD4+ T cells in HCMV reactivation or disease in haematopoietic stem cell recipients have not differentiated between the risk of developing HCMV viremia vs. the development of late-stage end-organ disease. Taking this into account, it was found that if patients were stratified into 3 groups: (i) those that could self-resolve infection, (ii) those that responded to treatment, and (iii) those that had recurrent infections and end-organ disease, this third group of patients had high levels of HCMV-specific CD8+ T cells, with persistently low levels of total CD4+ T cells and <1 cell/μl of blood of HCMV-specific CD4+ T cells 6 months post-transplant (Gabanti et al., 2015). However, in patients who developed late-stage HCMV gastro-intestinal disease, 6 out of 8 patients had levels of HCMV-specific CD4+ T cells above 1 cell/μl and had been viral DNA negative or at very low levels for 3 to 9 months before developing disease. This suggests that HCMV-specific CD4+ T cells numbers did not protect against the development of late-stage end-organ disease. It is important to note that all these patients were receiving immunosuppressive treatment, including low-dose steroids (methylprednisolone), at the time of diagnosis, and 6 out of 8 had received a transplant from a seronegative donor, which are known risk factors for CMV viremia (Gabanti et al., 2015).

#### Kinetics of Recovery of HCMV-Specific CD4+ T Cell Numbers and the Impact of Prophylaxis and Use of G-CSF on CD4+ T Cell Recovery

It has been theorized that HCMV reactivation causes activation of T cells, and this leads to an early expansion of T cells and faster reconstitution of T lymphocytes. In a study of 34 pediatric patients who underwent allogeneic BMT, the authors found that children with HCMV reactivation had a higher probability of reaching the 5th percentile of total CD4+ T cells of an age-matched healthy population (de Vries et al., 2000). This was also seen in a study of 201 adult R+ allogeneic non-T cell-depleted peripheral blood stem cell or bone marrow transplants (Hakki et al., 2003). CMV-specific CD4+ T cell responses, as measured by a lymphoproliferative response to CMV lysate, were significantly better in patients who developed breakthrough CMV antigenemia despite ganciclovir prophylaxis, vs. those who did not. However, a complicating factor is the use of high-dose steroids for treatment of graft- vs.-host disease (GVHD). When the subgroup of patients who developed breakthrough CMV antigenemia were analyzed, 100% of patients without GVHD had better recovery of the CMV-specific CD4+ T cell response compared to patients who received high-dose steroids. They thus concluded that high-dose steroids can override this inducing effect of breakthrough CMV antigenemia on the CMV-specific CD4+ T cell recovery (Hakki et al., 2003).

Emergence of CMV specific CD4+ T cell responses prior to the CD8+ T cell response has been shown, in a primary model of infection in solid organ transplant patients, to be associated with a lack of overt CMV disease (Rentenaar et al., 2000, 2001; Gamadia et al., 2003, 2004). In HSCT patients there is evidence that recovery of CD4+ T cells before CD8+ T cells may assist with priming the CD8+ T cell response via “licensing” of dendritic cells. Dendritic cell licensing refers to the phenomenon of upregulation of MHC class I and co-stimulators CD80/86 on dendritic cells after antigen presentation to CD4+ T cells via MHC class II and CD40-CD40L interactions have occurred. In this way, dendritic cells are able to present antigen to, and activate, CD8+ T cells, and this allows for tighter regulation of CD8+ T cell activation (Thaiss et al., 2011). In a study of 6 seropositive recipients of cord blood transplants, the appearance of CMV-pp65-specific CD4+ T-helper cells preceded an expansion of CMV-specific CD8+ T cells. When co-cultured with CD8+ T cells alone, these pp65-specific CD4+ T cells did not induce cytokine production by CD8+ memory T cells, but when done so in the presence of dendritic cells loaded with pp65, there was activation of these CD8+ memory T cells (Flinsenberg et al., 2015).

There has also been the suggestion that ganciclovir prophylaxis delays recovery of CMV-specific CD4+ (and CD8+) T cell responses possibly due to a decrease in viral replication, resulting in late-onset CMV disease (Li et al., 1994). This observation has led to the development of pre-emptive instead of prophylactic use of anti-viral drugs in patients. However, in a large study of 201 R+ allogeneic HSCTs (Hakki et al., 2003), there was no significant difference on CMV-specific CD4+ T cell recovery between patients who received prophylaxis vs. pre-emptive treatment with ganciclovir, the authors suggest this may be driven by subclinical reactivation of the virus despite ganciclovir treatment. The impact of anti-viral treatment resulting in decreased T-cell responses to HCMV stimulation has also been observed in pediatric allogeneic-HSCT patients. A study of 30 allogeneic-HSCT patients showed that the patients who received anti-CMV chemotherapy because of prolonged viremia had lower HCMV-specific CD4+ T cell numbers and delayed and depressed lymphoproliferative responses to HCMV stimulation (Guerin et al., 2010).

The use of peripheral blood stem cells (PBSCs) for transplantation improves survival in patients with high-risk hematological malignancies compared with the use of bone marrow (BM) as a stem cell source, because PBSC products from donors who have received G-CSF contain higher numbers of T cells and monocytes. However, PBSC recipients saw an increased incidence of early HCMV reactivation and delayed recovery of HCMV-specific immune responses, with a corresponding lower number of HCMV-specific CD4+ T cells (as measured by limiting dilution assay and CMV-specific cell lysis) in the stem cell product (Guerrero et al., 2012). This may be as a result of G-CSF administration to the donor, which is given in order to mobilize stem cells to migrate to the peripheral blood, but can also cause the reactivation of HCMV from latency. However, a subsequent study showed that although a reduced diversity of the TCRβ repertoire of CD4+ T cells was significantly correlated with HCMV (and EBV) reactivation, administration of G-CSF did not change this repertoire (Ritter et al., 2015). A recent study that measured the frequency of CD4+ T cells in recipients of PBSC grafts that produced IL-2, IFN-γ, or TNF-α in response to incubation with a HCMV lysate also did not find a deficiency in these cell responses compared to BM recipients (Waller et al., 2019). In fact, these recipients of PBSC grafts had faster T cell reconstitution, including more naïve CD4+ T cells. Therefore, more studies are required to determine if the apparent increased risk of HCMV reactivation with G-CSF use warrants a more cautionary use of this product.

Investigations of the recovery of CMV specific CD4+ T cells in HSCT patients demonstrated that there are different kinetic patterns that result in the recovery of the CD4+ T cell response: (i) rapid expansion of IFN-γ secreting T cells within the first week after initiation of pre-emptive therapy concomitant with rapid clearance, (ii) early expansion of a lower magnitude than that seen in rapidly cleared episodes, and (iii) an inconsistent or lack of expansion associated with persistent CMV DNAemia (Tormo et al., 2010b). The reconstitution of HCMV-specific CD4+ T cells can also be stratified by donor and recipient serostatus—recovery is fastest in D+/R+, followed by D–/R+, and is slowest in D+/R– populations (Lilleri et al., 2008a). In fact, in D+/R+ patients, it appears that the reconstitution kinetics of HCMV-specific CD4+ T cells are the same as HCMV-specific CD8+ T cells (Foster et al., 2002). It is important when interpreting these results to remember that reconstitution of CMV specific CD4+ T cells is not equivalent to recovery of a fully functional CMV specific CD4+ T cell response. Measuring whether there is a lymphoproliferative response to CMV antigens is possibly more reflective of the actual ability of the T cells to prevent HCMV reactivation and disease. Early studies in allogeneic bone marrow transplant patients showed that up to 30% of recipients with a lack of a CMV-specific CD4+ lymphoproliferative responses by day 120 post-transplant develop CMV disease (Krause et al., 1997). When HCMV-specific CD4+ T cells in pediatric allogeneic HSCT recipients were examined for both IFN-γ and proliferative responses, there was first a recovery of the IFN-γ response before the proliferative response (Guerin et al., 2010). This is also seen in primary HCMV infection, where development of the lymphoproliferative response to HCMV is delayed compared to the development of CD4+ and CD8+ IFN-γ-producing T cells (Fornara et al., 2016).

#### Surface Markers of HCMV-Specific CD4+ T Cells in HSCT Recipients

Alongside measuring HCMV T-cell reconstitution in HSCT recipients, some studies have assessed whether HCMV-specific CD4+ T cells which are polyfunctional, measured by an ability to produce both IFN-γ and IL-2 in response to HCMV, are more likely to be protected from HCMV reactivation (Lilleri et al., 2008a). IL-2 is a cytokine which can have multiple effects on CD4+ T cell immune responses, including modulating the development of T cells into memory subsets. It signals to the T cell via binding to the IL-2 receptor, a complex consisting of three chains, termed α (CD25), β (CD122), and γ (CD132) (Liao et al., 2011). Increased risk of HCMV reactivation is associated with reduced numbers of CD4+CD25^high^ cells, and a study of 99 HSCT recipients found that numbers of CD4+CD25^high^ but not CD4+ T cells was an independent factor for risk of CMV reactivation (Jaskula et al., 2015). The expression of other functional markers on HCMV specific CD4+ T cells have also been studied in HSCT and SOT patients. Patients with PCR-positive reactivations after HSCT were found to have more frequent occurrences of CD4+ T cells with degranulation markers CD107a and co-stimulatory molecule CD40L (Krol et al., 2011). The frequencies of appearance of these markers corresponded with a higher antigen load. This subpopulation of CD4+ T cells was previously described to be MHC class II-restricted cytotoxic T cells in primary disease in SOT patients (Gamadia et al., 2004; van Leeuwen et al., 2004; van de Berg et al., 2008). The typical phenotype of CMV specific CD4+ T cells in healthy people has been described in the introduction of this review and include the loss of co-stimulatory molecules CD28 and CD27. This phenotype is also observed in SOT patients (van Leeuwen et al., 2004, 2006; Dirks et al., 2013; Burton et al., 2018, 2019) and is now used to predict CMV infection history in transplant patients where the use of serology is unreliable (Burton et al., 2018, 2019). The loss of PD-1 is also observed in these patients (Dirks et al., 2013). These HCMV-specific CD4+ T cells tended to show impaired production of IL-2 for first 6 months following HSCT, but the ratio of IL-2/IFN-γ production then increases with time post-transplant (Pourgheysari et al., 2009), suggesting a conversion from an effector memory to a central memory phenotype. HCMV reactivation has also been demonstrated to cause a contraction of the TCRβ diversity. An investigation of the CD4+ T naïve population from 7 HSCT recipients that had HCMV reactivation showed that these patients had a progressive loss of CD31+CD4+ T_naï*ve*_ cells. CD31+CD4+ T cells are enriched in new thymic emigrants, and a loss of this population suggests that there is thymic compromise in patients that reactivate HCMV (Suessmuth et al., 2015). HCMV reactivation was also associated with significant expansion of CD8+ T cells, resulting in an inversion of the CD4:CD8 ratio in HCMV reactivating patients (Suessmuth et al., 2015). The authors cited previous studies which show that HCMV can infect thymic epithelium and activated and effector T cells can directly infiltrate and damage the thymus (Mocarski et al., 1993).

### CD4+ T Cells and Adoptive Transfer Therapies for HCMV Disease in Transplant Patients

Since the initial trial of donor T cell infusion in 1995 (Walter et al., 1995), multiple phase 1 and 2 clinical trials of adoptive T cell therapies in HSCT recipients have been performed (reviewed in Meesing and Razonable, 2018; van der Heiden et al., 2018a; Girmenia et al., 2019). In SOT recipients, the challenge of autologous adoptive T cell therapy is to be able to generate a sufficient number of CMV-specific T cells from the immunosuppressed recipients. Multiple case reports performed in mostly lung transplant recipients appear to have shown potential (Brestrich et al., 2009; Holmes-Liew et al., 2015; Pierucci et al., 2016), though there has been just one clinical trial of autologous CMV-specific T-cell therapy in SOT recipients so far (Smith et al., 2018).

In the initial published trial of adoptive immunotherapy (Walter et al., 1995), clones of CMV-specific CD8+ cytotoxic T cells were infused into 14 allogeneic bone marrow transplant recipients. There was reconstitution of CMV-specific T cell cytotoxicity in all patients, but this activity subsequently declined in patients that were deficient in CMV-specific CD4+ T cells, suggesting that CD4+ T cells were crucial in the maintenance of the CMV-specific T cell response. There was a change of approach in subsequent trials of adoptive immunotherapy, and preparation of the T-cell infusions involved pulsing donor dendritic cells with CMV antigen, then co-culturing with PBMCs and subsequently selecting for CMV-specific T cells, resulting in infusions containing CD8+ and CD4+ CMV-specific T cells. Though not all the trials evaluated if the infusions consisted of more CD8+ or CD4+ T cells, in those that did, there appears to be a predominance of CD4+ T cells. As the use of adoptive immunotherapy was pursued, subsequent clinical trials modified the protocol to stimulate mononuclear cells isolated from peripheral blood of the donors up to 4 times with CMV antigen, and the resultant proliferation of CMV-specific T cells was mostly CD4+ dominant. Following infusion, clinically there was clearance of viremia in 5 out of 7 patients, although it should be noted that these patients had only low to moderate levels of viremia (Einsele et al., 2002). This is supported by a few later studies (Leibold et al., 2012; Albiero et al., 2016) that looked at the CD4+/CD8+ ratio within T-cell lines isolated for IFN-γ production in response to pp65 stimulation—in one study, up to 90% of the T cells were found to be CD4+ (Leibold et al., 2012). The CMV specific CD4+ T cells that are infused perform better when they replicate the phenotype and functionality of effective CD4+ T cell responses against CMV disease in transplant patients. More recently the functional and phenotypic characteristics of HCMV peptide pool-generated antigen-specific CD4+ T cells used for CMV T cell therapy has been assessed (Hammoud et al., 2013). This study proposes that it is important to generate polyfunctional CMV specific CD4+ T cells that are both directly anti-viral but can also support CMV specific CD8+ T cell responses to improve the efficacy of adoptive CMV specific T cell therapy in patients. In the majority of these T cell therapy trials, the CMV antigens used to generate the CMV-specific T cell lines were derived from the tegument protein pp65. Interestingly, one study compared using pp65 vs. IE1 antigens as stimulant (Albiero et al., 2016), and found that there was a much higher degree of expansion with IE1 (1- to 961-fold) than pp65 (1-to 33-fold), and that there was a greater expansion of CD4+ T cells exhibiting a T_naï*ve*_ stem cell phenotype (CD62L+CD45RA+) on stimulation with IE1 as compared to pp65. This demonstrates that when developing adoptive T cell therapy for CMV disease it is important to consider generating polyfunctional CD4+ T cells specific to multiple CMV antigens.

Of recent interest has also been the use of stored CMV-specific T cells from third-party donors for T cell therapy. This involves generating virus-specific T cell lines (VST) from pre-selected donors and expanding these VSTs *ex vivo*. These T cells are then cryopreserved, and, when needed for patients with refractory viremia, a VST from a HLA-matched donor can be used “off-the-shelf.” The advantage of such an approach over using VSTs from a specific donor is that it eliminates the usual 2–3 week waiting period needed to generate a VST. A multi-center trial involving 23 patients with refractory CMV infection showed that 17 of these patients responded to VST infusion, although the authors of this study could not identify a correlation between CD4+ T cell numbers or percentage in the infused line with the strength of clinical response (Leen et al., 2013). A later study involving 30 allogeneic HSCT recipients with persistent or recurrent HCMV, EBV or adenovirus infections tracked the subpopulations of T-lymphocytes in these patients up to 1 year post-infusion, and found that within the CD4+ T cell memory subset, effector memory T cells were dominant throughout follow-up (Withers et al., 2017). The percentage of CD4+ T cells in these infusions ranged from 15 to 85%, but the authors made no comment on an association of CD4+ T cell proportion with successful treatment. A third study consisted of 8 HSCT recipients who received third party donor infusions of HCMV-specific CD8+ T cells. In this study it was thought that HCMV-specific CD4+ T cells were not essential for the activity of these CD8+ T cells. However, 3 of the 8 patients died, and only one treated donor successfully expanded the transferred CD8+ T cell population (Neuenhahn et al., 2017). Thus, more studies are required to determine if HCMV-specific CD4+ T cells are essential for successful treatment of persistent or recurrent HCMV disease by third party donor lymphocyte infusions.

### CD4+ T Cells and Congenital HCMV

The risk of transmission of CMV from mother to fetus, resulting in congenital CMV infection, is highest in primary infection in the mother, with reported ranges of approximately 40% (Fowler et al., 1992). However, transmission of CMV to the fetus can also occur in mothers who are seropositive, albeit at much lower rates (Kenneson and Cannon, 2007; Britt, 2015). These were initially thought to occur as a result of reactivation of latent virus, although more recent studies have suggested that infection with a serologically distinct strain of HCMV may be a cause as well (Ross et al., 2010; Yamamoto et al., 2010).

The kinetics of the development of an antibody response during primary HCMV infection in pregnant vs. non-pregnant women appear to be comparable (Revello et al., 2006), but pregnant women having a primary infection appear to have a decreased CD4+ lymphoproliferative response to CMV lysate and IL-2 production for at least 9 months after infection (Fornara et al., 2011). Mothers that do not transmit CMV to the fetus are more likely to have an earlier and higher lymphoproliferative response of CD4+ T cells to HCMV (Revello et al., 2006; Fornara et al., 2016), with some observations that the CD4+ response develops earlier than the CD8+ lymphoproliferative response (Lilleri et al., 2007a). The CMV-specific CD4+ T cells of these non-transmitting mothers also had higher percentages of IL-7Rpos (Mele et al., 2017), CD45RA+ (Fornara et al., 2011, 2016), and IL-2 (Fornara et al., 2016). When compared with healthy sero-negative pregnant mothers, the CD4+ T cells of sero-positive pregnant women had higher levels of IFN-γ and TNF-α production in response to exposure to CMV antigen, but this response was less than in healthy, non-pregnant seropositive females (Fujikawa et al., 2003). In fact, an examination of 44 pregnant women with primary HCMV infection showed that most of these IFN-γ-producing T cells were CD4+ (Fornara et al., 2017).

Decreased cytokine production following stimulation with CMV antigens is also seen in infants with congenital CMV. An analysis of seven infants with congenital CMV infection showed a lack of production of IFN-γ, IL-2, and IL-4 from CD4+ T cells on exposure to pp65-derived peptide (Hayashi et al., 2003). Other early studies made the observation that symptomatic children with congenital CMV had higher percentages of CD4+ T cells that produced IFN-γ and TNF-α in response to CMV antigen, though there was a limitation of small sample sizes (Numazaki et al., 2002; Fujikawa et al., 2003), and a later study of the response of CD4+ T cells from congenitally infected infants showed they had a reduced polyfunctional response (defined as ≥2 out of CD107, MIP1β, IFN-γ, and/or IL-2) to pp65 antigen (Gibson et al., 2015).

A comparison of congenitally infected neonates and their mothers showed that neonatal sera contained significantly higher levels of IL-8 when compared with their mothers, and also had increased levels of IL-2, IL-12, and IFN-γ with a corresponding lack of IL-4, suggesting a predominantly T helper 1 response (Hassan et al., 2007). There may also be extrapolations that can be made from studies of HIV-positive mothers co-infected with HCMV. A maternal CD4+ T cell count of <200 cells/ul is associated with higher risk of transmission to the fetus (Gantt et al., 2016). Retrospective studies of infants born to HIV-positive mothers showed that, if their mothers received full anti-retroviral prophylaxis, they had higher CD4+ T cell counts (Mania et al., 2013) and were less likely to have congenital CMV (Guibert et al., 2009).

A large Swedish study of infants up to 2 years of age with congenital CMV infection found that they had CMV-specific CD4+ T cell responses (measured by IFN-γ) that were inferior compared to adults during the first 3 months of age, though this difference was not significant by the age of 24 months (Lidehall et al., 2013). This was in contrast to the CD4+ T cell responses in 8 adults with primary CMV infection, which was high initially and then subsequently decreased. This increase in CMV-specific CD4+ T cells appears to be approximately linear (Chen et al., 2016). The slower increase of CD4+ T cell function may explain the longer duration of viral shedding seen in neonates and children (Tu et al., 2004; Cannon et al., 2011), and illustrates the important role CD4+ T cells play in controlling CMV disease. In addition to causing a slower increase of fetal CD4+ T cells, CMV infection *in utero* also appears to cause an oligoclonal expansion of CD4+ T cells in the infected newborn. Higher frequencies of CD27-CD28-CD4+ T cells were detected in newborns with congenital CMV, with decreased expression of CCR7, IL-7R and increased expression of CD57 and the transcription factor T-bet and chemokine receptor CCR5, indicating Th1 and Tc1 phenotypes. They also had a higher expression of the PD-1 inhibitory receptor, a similar profile to that seen in exhausted T lymphocytes (Huygens et al., 2015).

The importance of CD4+ T cells to generate a sustained and protective response to CMV is also seen in vaccine studies. In the rhesus model of CMV, rhesus macaques that received CD4+ T-cell-depleting antibody had fetal loss or infant rhCMV-associated sequelae (Bialas et al., 2015). A phase 2 clinical trial for a gB-based vaccine with MF59 adjuvant showed an efficacy of 50% (Pass et al., 2009), and subsequent analysis of the immune response showed that there was not only an increase in antibody production but there also an increase in gB-specific CD4+ T cell proliferation and IFN-γ production after vaccination (Sabbaj et al., 2011), suggesting that, just like in primary infection (Gamadia et al., 2003), the formation of effector memory CD4+ T cells was needed for an effective and sustained immune response to CMV.

### CD4+ T Cell Lessons From Murine Models

Whilst the many human studies described in this review have illustrated the essential role CD4+ T cells play in resolving CMV disease, there are limitations to these studies. The use of mouse models can help to inform our understanding of the mechanisms and function of CD4+ T cells in CMV disease. During acute MCMV infection in mice, the CD4+ T cell response peaks early and then contracts sharply to very low levels, and is dominated by high frequencies of IFN-γ and TNF-α double-producing CD4+ T cells (Arens et al., 2008; Walton et al., 2008). These MCMV-specific CD4+ T cells accumulate in the spleen and lungs and produce multiple cytokines—IFNγ, TNF, IL-2, IL-10, and IL-17 (Arens et al., 2008). In the lungs of infected mice, nodular inflammatory foci form around infected cells, which contain CD8+ and CD4+ T cells and exert viral control via IFN-γ and perforin (Lueder et al., 2018). However, in the context of suppressing viral reactivation, CD4+ T cells are not as essential, as experiments in a B-cell deficient mouse model have established a hierarchy of CD8+ T cells being more crucial to suppressing viral reactivation compared to CD4+ T cells (Polic et al., 1998), with viral control and expansion of these MCMV-specific CD4+ T cells being dependent on CD27-CD70 co-stimulation (Welten et al., 2013). There is also evidence for cytolytic activity of CD4+ T cells in MCMV model. MCMV-specific CD4+ T cells that had high levels of granzyme B expression were able to lyse infected target cells in the BALB/c mouse liver. In addition, CD4+ T cell epitope vaccination of immunocompetent mice reduced MCMV replication in the same organs where this cytotoxic activity was seen (Verma et al., 2015).

#### Approaches to Examining the Role of CD4+ T Cells in MCMV Infection

There have been multiple approaches to interrogating the role of CD4+ T cells in the control of MCMV infection. The first approach involves depletion of CD4+ T cells. This was initially achieved through injecting mice with anti-CD4+ (L3T4) antibodies. Early studies using anti-CD4+ monoclonal antibodies to deplete CD4+ T cells showed that these BALB/c strain of mice had delayed clearance of replicating virus, but were still able to generate protective CD8+ effector T cells and restrict viral replication to the acinar cells of the salivary glands (Jonjic et al., 1989). This finding was repeated in a later experiment using a different mouse strain, C57BL/6, where mice depleted of CD4+ T cells were unable to control chronic viral replication in the liver and salivary glands (Walton et al., 2008). Subsequently, it was demonstrated that MHC class I and II expression was detectable only at low levels in salivary gland cells and that antigen-presenting cells in the salivary gland were deficient in cross-presentation to CD8+ T cells, thus control of MCMV replication in the salivary gland was likely to be due to CD4+ T cells that had been selectively induced by antigen-presenting cells in the salivary glands (Walton et al., 2011a). These MCMV-specific CD4+ T cells produce IL-10, which in turn is induced by IL-27, and these cytokines promote persistence of MCMV in the salivary glands (Humphreys et al., 2007; Wehrens et al., 2018).

Another approach involved generating knockout mouse models—CD4^−/−^ and MHC II^−/−^. One major difference between these two lines is that CD4^−/−^ mice are able to generate isotype-switched antibody responses. This is achieved via a population of CD8- CD4- T cells that are capable of adopting some of the function of T_helper_ cells, such as mediating antibody class switching (Locksley et al., 1993; Rahemtulla et al., 1994) and supporting somatic hyper-mutation and affinity maturation of germinal center B cells (Zheng et al., 2002). There also exists a population of MHCII-restricted T cells that are misdirected into the CD8 lineage (Matechak et al., 1996; Tyznik et al., 2004). In contrast to the mice depleted with anti-CD4+ T cell antibodies, when CD4^−/−^ mice were infected with MCMV, these mice were able to clear viral infection in all organs, albeit at a slower rate (of 200 to 400 days post-infection) than wildtype controls (Walton et al., 2011a). A possible reason for this difference is that the viral loads in the organs of the CD4^−/−^ mice were observed for much longer periods than the earlier studies. When MHC II^−/−^ mice were infected with MCMV, they were not able to eliminate viral replication. As MCMV-specific antibodies were previously shown to inhibit viral dissemination during MCMV infection, the authors surmised that the inability to generate isotype-switched antibody responses was the likely reason that CD4^−/−^ but not MHC II^−/−^ mice were able to halt active MCMV replication (Jonjic et al., 1994; Wirtz et al., 2008).

It thus appears that CD4+ T cells are not essential to elimination of actively replicating MCMV. To examine if CD4+ T cells provide assistance to CD8+ T cells in clearance of replicating virus, CD4^−/−^ mice were infected with MCMV, and the percentage of CD8+ T cells that recognized various MCMV epitopes were measured at multiple time points post-infection. The results showed that only accumulation of the late-appearing IE3-specific CD8+ T cells was substantially impaired, suggesting that the help that CD4+ T cells provide to CD8+ T cells is limited to assisting in the expansion of only a limited subset of MCMV-specific CD8+ T cells (Snyder et al., 2009). A caveat of interpreting this result was that only very limited epitopes (M45, M38, m139, and IE3) were tested, and with the knowledge that a large repertoire of epitopes are recognized by T cells, perhaps more extensive testing needs to occur. The CD4+ T cell help provided via MHC II expression is also needed to maintain a stable CD8+ T cell memory pool, although ongoing lytic viral replication is partially able to provide this assistance as well. When splenic CD8+ T cells from CD4-deficient MHC II^−/−^ mice that had been chronically infected with MCMV were transferred into mice that were then infected with MCMV, the CD8+ T cells from MHC II^−/−^ mice proliferated much less vigorously than CD8+ T cells from wildtype mice (Walton et al., 2011b).

A third approach involves using adoptive transfer techniques, which can help to inform the equivalent adoptive transfer T cell therapies employed in transplant patients. Early studies of transfer of CD4+ T cells into irradiated Balb/c mice that were subsequently infected with MCMV showed that CD4+ T cells were not able to prevent viral replication in the lungs (Reddehase et al., 1985, 1987), spleen or adrenal glands (Reddehase et al., 1988). Later studies using the same murine system also demonstrated that controlling CMV mediated lung disease in treated mice required CD8+ T cells rather than CD4+ T cells (Steffens et al., 1998; Podlech et al., 2000). However, when adoptive transfer was performed in severe combined immunodeficiency (SCID) mice, CD4+ T cells were able to prevent viral dissemination in the brain (Reuter et al., 2005). Overall, therefore, CD4+ T cells appear to be essential only for control of viral replication in the specific organs in the mouse model.

#### Caveats to Interpreting MCMV Models

Limitations exist in extrapolating the findings in murine models of cytomegalovirus infection, due to the underlying differences between murine CMV infection and HCMV (Lemmermann and Reddehase, 2016). In an early mouse model of adoptive immunotherapy, transfer of CD4+ T cells into irradiated and MCMV-infected mice did not reduce viral titers in the lungs, spleen nor adrenal glands of these mice. In contrast, transfer of CD8+ T cells had significant reductions in viral titers (Reddehase et al., 1988). When graded numbers of CD4+ T cells were transferred with a constant number of CD8+ T cells, there was no difference to viral titers either (suggesting no helper effect). However, as already discussed it is clear that in the case of HCMV infection CD4+ T cells are a necessary component of CMV T cell therapy.

There have thus been attempts to create a “humanized” mouse model of CMV infection, by generating an immune deficient mouse with a mutation in IL-2 receptor γ-chain locus (IL-2γc ^−/−^) that is severely impaired in generating mouse B, T and NK cell lines (reviewed in Shultz et al., 2012; Crawford et al., 2015). When these mice were engrafted with human haematopoietic progenitor cells, they were able to reconstitute monocytes, macrophages and limited T-cells. This model was further refined by reconstituting these mice with human fetal bone marrow, liver and thymus tissue (Covassin et al., 2013). Latent infection of these mice were able to induce generation of central and effector memory HCMV-specific T-cells and produce HCMV-specific IgM and IgG neutralizing antibodies (Crawford et al., 2017). Adoptive transfer of CD4+ T cells in such a model has shown that these CD4+ T cells did not have an anti-viral effect on their own, but when co-administered with CD8+ T cells, they appeared to enhance the anti-viral efficacy of CD8+ T cells and significantly decreased viral titers in the spleen and lungs (Thomas et al., 2015).

## Conclusions

It is clear that there is increasing evidence to show that CD4+ T cells play a significant role in anti-viral immunity to HCMV. The virus has evolved immune evasion mechanisms to target the MHC class II antigen presentation pathway, the method by which CD4+ T cells TCR recognize presented viral peptides triggering cell activation and anti-viral functions. Following primary infection, there is development of a CMV-specific CD4+ T cell population that persists in the T cell repertoire of healthy adults, suggesting that this population of T cells is required for a healthy immune response to control periodic episodes of viral reactivation over a life time of the infected host. In both SOT and HSCT recipients, the presence of CMV-specific CD4+ T cells are highly associated with lower risks of developing CMV disease by reactivation of latent virus, and conversely, the lack of this population of T cells herald a higher likelihood of developing recurrent CMV viremia and end-organ disease.

However, many questions still remain unanswered. The majority of studies referenced in this review show an association of the presence of CMV-specific CD4+ T cells with protection from disease, but few have attempted to explain the mechanism of how this occurs. Attempts to interrogate how they exert their effects have mostly been limited to demonstrating presence of cytokines and activation markers on these CD4+ T cells as a response to exposure to CMV antigens. More work has been done in the murine model on attempting to elucidate this mechanism by generating various knockout mouse models, but interpretations from these models are limited by the apparent greater dispensability of CD4+ T cells in control of MCMV disease. As such, more work needs to be done to investigate this, with the possibility of using this knowledge to further refine techniques for adoptive therapies or vaccine studies. In addition, it is also clear that current techniques of measuring CD4+ T cell responses do not provide a complete picture of the contribution of CD4+ T cells to the immunological response to CMV. Methods that assess effector function more accurately, such as anti-viral assays, may provide a more nuanced prediction of developing CMV-related disease, and allow clinicians to tailor anti-viral therapies better.

## Author Contributions

EL wrote the initial draft of this manuscript. SJ, EL, and MW revised the manuscript. All authors approved the manuscript for publication.

## Conflict of Interest

The authors declare that the research was conducted in the absence of any commercial or financial relationships that could be construed as a potential conflict of interest.
